# Three-Dimensional Bioprinting and Rose-Inspired Medical Applications

**DOI:** 10.3390/biomimetics11030164

**Published:** 2026-03-01

**Authors:** Hsiuying Wang

**Affiliations:** Institute of Statistics, National Yang Ming Chiao Tung University, Hsinchu 300093, Taiwan; wang@stat.nycu.edu.tw

**Keywords:** 3D bioprinting, bioink, medical application, rose, plant

## Abstract

Three-dimensional (3D) bioprinting is an advanced additive manufacturing technology that utilizes bioinks composed of living cells and biomaterials to construct tissue-like structures for a wide range of medical applications. This paper reviews key applications, including tissue engineering, organ modeling and printing, drug testing and development, disease modeling, cosmetics and chemical testing, regenerative medicine, and personalized medicine. In parallel, biomimicry of natural plant architectures offers powerful opportunities for innovation in biomedical material design. Among these, the rose stands out for its intricate hierarchical geometry, which provides not only aesthetic appeal but also exceptional mechanical resilience. Incorporating rose-inspired structural elements into 3D-bioprinted medical constructs can significantly enhance mechanical strength, flexibility, and surface adaptability. This review also highlights plant- and rose-inspired approaches in medical applications and outlines the potential of rose-inspired 3D bioprinting to advance the design of functional and biomimetic tissue models. Nature provides a rich source of inspiration for biomimetic design, and translating biological principles into engineering solutions can contribute to sustainable technological development aligned with the Sustainable Development Goals (SDGs). In this regard, roses and other plant systems offer valuable structural and functional inspiration for advancing 3D bioprinting in medical applications.

## 1. Introduction

Three-dimensional (3D) printing is a rapidly emerging additive manufacturing technology with broad applications across industries and biomedical fields [1,2]. It involves a layer-by-layer fabrication process, where material is sequentially deposited to build objects based on a digital geometric model [3]. In general, the 3D printing process consists of two main stages: (1) converting digital design data into a printable format, and (2) fabricating the object layer by layer, either by moving a print head or a build platform, or through selective curing or binding techniques, depending on the specific printing technology [4,5]. Key advantages of 3D printing include design flexibility, mass customization, material waste reduction, the ability to fabricate complex structures, and rapid prototyping capabilities [6]. It also enables on-demand production with high efficiency and cost-effectiveness.

Three-dimensional printing has emerged as a prominent manufacturing technique in healthcare and medicine, supporting a wide array of applications such as dentistry, tissue engineering, regenerative medicine, engineered tissue models, medical devices, anatomical replicas, and drug formulation [7]. Three-dimensional printing in pharmaceuticals has advanced rapidly, contributing to the development of customized medical solutions, cost reduction, enhanced production efficiency, expanded access to localized manufacturing, and improved interdisciplinary collaboration [8]. Compared to traditional methods, it offers key advantages in personalized drug manufacturing, enabling rapid, small-scale production and the fabrication of complex drug structures with tailored release profiles [9].

The loss of organs and tissues due to disease or injury has driven the search for therapies that restore function and reduce dependence on transplants. Regenerative medicine is an interdisciplinary field that combines engineering and life sciences to repair or replace damaged tissues and entire organs by harnessing the body’s natural healing processes [10]. Three-dimensional bioprinting has become a major enabler by allowing the precise placement and organization of biomaterials together with patient- or donor-derived cells. It has played a crucial role in advancing the creation of in vitro tissue models, preclinical implant prototypes, and organ-like constructs [11]. A key goal in regenerative medicine is to leverage the biological potential of stem cells within structurally complex scaffolds or microenvironments. Recent progress in bioprinting has enabled the precise printing of stem cells while maintaining their high viability, functional integrity, and pluripotent capabilities. This makes bioprinting a highly promising tool for regenerative medicine, supporting applications such as guided stem cell differentiation, tissue development, and the formation of vascular networks [12].

**Figure 1:**
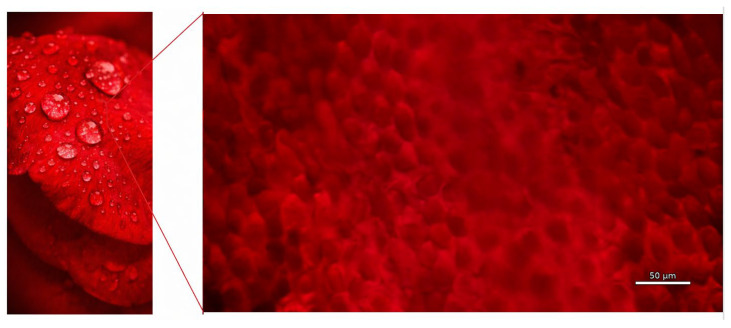
Microscale surface structures of a rose petal (the figure was created by the author).

Nature has long been a source of inspiration in technological development, including advancements in 3D bioprinting. Many biological surfaces, like lotus leaves, butterfly wings, rose petals, and shark skin, possess unique micro- and nanostructures (Figure 1) that demonstrate properties and functions beyond those achievable by traditional engineering [13].

Biomimetic graphene, inspired by structures like rose petals and butterfly wings, combines graphene’s exceptional properties with nature-inspired designs, offering promising applications in research and industry [14]. Inspired by rose petal micropapillae, a superhydrophobic surface with micro-bulge structures was created via laser direct writing on a tin–bronze alloy [15]. The surface demonstrated excellent water repellency, and this low-cost biomimetic approach can enhance the performance of hydrostatic bearings. Inspired by natural surfaces like lotus leaves, rose petals, and lizard skin, self-cleaning materials use superhydrophobic or superhydrophilic properties to repel dirt and water [16]. These surfaces are rough and have nano/microstructures with low surface energy. In particular, rose petals exhibit high-adhesion and a Wenzel-type superhydrophobic surface. The petal effect refers to their ability to firmly retain water droplets, preventing them from rolling off even though the surface is hydrophilic [17]. This unique adhesive property holds significant potential for medical applications, including controlled fluid retention, enhanced cell adhesion, and moisture-preserving wound dressings.

Three-dimensional bioprinting has demonstrated significant potential across a wide range of medical applications. This review focuses on plant- and rose-inspired approaches, highlighting their roles in enhancing biomedical material design and functional performance. The reviewed literature is selected based on relevance to 3D printing, bioprinting, and plant-inspired design, and is organized around key themes including technical principles, biomedical applications, and biomimetic design strategies. The scope encompasses conventional 3D printing, bioprinting technologies, and plant- and rose-inspired architectures, to support conceptual understanding, comparative discussion, and application-oriented insights into recent advances in 3D bioprinting.

## 2. Three-Dimensional Printing Principle

### 2.1. Traditional 3D Printing

In general, 3D printers fall into several categories: (1) printers that extrude molten or semi-liquid materials; (2) those that cure light-sensitive resins; (3) printers that fuse or bind powder particles; and (4) devices that layer and bond sheets of paper, plastic, or metal [4]. These traditional 3D printing technologies, including stereolithography (SLA), fused deposition modelling (FDM), selective laser melting (SLM), electron beam melting (EBM), and laminated object manufacturing (LOM) [18], each represent a distinct method for creating 3D objects layer by layer.

SLA is an additive manufacturing technique that creates 3D objects by using UV light to cure liquid photopolymer layer by layer [19]. The process is based on photopolymerization, in which light activates the bonding of molecular chains, transforming the resin into solid structures. FDM is a widely used 3D printing technique that forms 3D structures by melting and extruding plastic filament layer by layer. The process starts with a digital model created using 3D design software, which is then translated by the printer into a physical object through sequential material deposition [20]. It is known for its low cost, ease of use, and suitability for prototyping and educational applications.

SLM is a 3D printing technology that uses a high-powered laser to fully melt metal powder, which then cools and solidifies layer by layer into dense, complex parts [21]. It eliminates the need for molds, reducing production time and costs, and is widely used in aerospace, medical, and industrial fields for its precision and material strength. EBM is an advanced metal additive manufacturing technology that uses an electron beam to fully melt metal powder layer by layer in a vacuum chamber to build complex parts. Operating in a vacuum prevents oxidation of reactive metals like titanium and eliminates collisions with gas molecules, ensuring the production of strong, high-quality metal parts [22]. LOM builds 3D objects by stacking and bonding layers of material, typically paper, plastic, or metal foil, and then cutting each layer to shape using a laser or blade. Unlike many other 3D printing methods, LOM machines provide an open workspace and can efficiently process non-toxic, highly filled sheet materials at high speeds [23].

### 2.2. Bioprinting

#### 2.2.1. Categories

In addition to traditional 3D printing methods, 3D bioprinting is an advanced biofabrication technique that integrates living cells and biomaterials to construct functional biological structures with high spatial precision, aiming to replicate native tissues or organ-like architectures [24]. These materials, known as bioinks, are gel-like substances made from one or more cell-friendly hydrogel precursors, usually mixed with the required cell types, and are designed to solidify in ways suited to various bioprinting methods for creating tissue structures [25,26]. It involves the computer-aided deposition of both living and non-living materials in predefined 2D or 3D patterns to recreate biological structures [27]. Three-dimensional bioprinting is primarily applied in tissue engineering, regenerative medicine, and drug testing.

**Figure 2:**
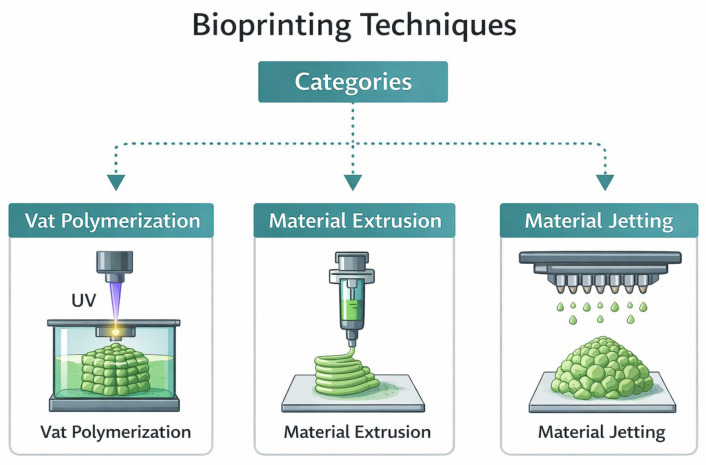
Three main categories of bioprinting, including vat polymerization, material extrusion, and material jetting.

This emerging technique offers high reproducibility and precise control over the fabrication of constructs through automation. Three-dimensional bioprinting builds on similar core principles of additive manufacturing but adapts them for use with bioinks, materials containing living cells and biological molecules. Bioprinting techniques are broadly classified into three main categories: vat polymerization, material extrusion, and material jetting [27] (Figure 2).

Vat polymerization is a 3D printing technique that uses photopolymerization to solidify liquid resin in a vat, building the object layer by layer into a 3D structure [27]. In this method, a photopolymer resin is stored in a vat and cured using light, usually ultraviolet (UV). The resin is selectively hardened into layers by either a laser beam (SLA method) or a projected light pattern (Digital Light Processing (DLP) method), forming the object one layer at a time. These two methods of light-based curing in vat polymerization are known as SLA and DLP, respectively.

SLA printing uses a laser beam that moves in a raster scan pattern, curing the resin line by line until an entire layer is completed [28]. The laser moves back and forth across the surface of the resin in a raster scan pattern. As it moves, the laser draws out the shape of the layer by curing the resin line by line. Once the entire pattern for that layer is finished, the build platform lowers slightly, and the process repeats for the next layer, eventually building up the full 3D object. DLP, on the other hand, uses a digital light projector to flash an entire layer pattern at once, curing it more quickly than SLA [29]. DLP offers several advantages, including low equipment cost, high resolution, fast printing speed, and enhanced efficiency [30,31,32]. Vat polymerization, using SLA or DLP techniques, is adapted for bioprinting by employing biocompatible photopolymers and lower-energy light sources to avoid damaging cells. This method offers high resolution and is particularly suited for printing detailed structures such as vascular networks or skin scaffolds.

Material extrusion is one of the most commonly used bioprinting methods. Among the various bioprinting techniques, extrusion-based 3D bioprinting was one of the earliest developed and remains widely used for fabricating 3D tissue constructs [33]. This method operates by extruding bioink, typically from a syringe, through a nozzle using either mechanical or pneumatic force. This process forms continuous microfilaments that are deposited onto a receiving substrate and assembled layer by layer into the intended 3D structure. The substrate may be solid, such as a culture dish, liquid like a growth medium, or composed of gel-derived materials. The nozzle’s path is generally controlled by software based on preconfigured digital models. Critical parameters, including temperature, nozzle diameter, extrusion pressure, movement speed, extrusion rate, and path spacing, significantly influence the fidelity and functionality of the final bioprinted construct [34]. This technique allows for the printing of highly viscous biomaterials and is widely applied in the fabrication of tissue scaffolds, skin equivalents, and cartilage. The flexibility of extrusion systems makes them suitable for incorporating a variety of cells and bioactive molecules.

Material jetting methods include inkjet, microvalve, and laser-assisted bioprinting. These techniques dispense small droplets of bioink in a controlled manner to build up the desired structures [35]. In particular, laser-assisted bioprinting uses laser pulses to propel high-density, cell-laden droplets onto substrates with exceptional precision and resolution, and maintains high cell viability [36]. The jetting-based bioprinting offers precise, contactless deposition of sub-nanoliter bioink droplets in a drop-on-demand fashion. This high-resolution approach is especially advantageous for applications that require spatial accuracy and gentle cell handling. Its versatility has enabled advances across various biomedical fields, including tissue engineering, regenerative medicine, wound healing, drug development, and cell transfection. As bioprinting technologies continue to evolve, jetting-based methods are poised to play an increasingly important role in fabricating functional, high-fidelity biological models and therapies [37].

#### 2.2.2. Bioink

Three-dimensional bioprinting is an innovative additive manufacturing technique. Despite its promise, a major hurdle to its clinical and preclinical adoption remains the development of bioinks that simultaneously offer excellent printability, mechanical integrity, biodegradability, and biological functionality compatible with living cells. That is, a material suitable for 3D printing must fulfill several essential requirements. First, it should exhibit adequate biocompatibility to support cell survival, proliferation, and function. Second, appropriate printability is required, including suitable rheological properties that allow stable extrusion or deposition while maintaining shape fidelity after printing. Third, the material should possess tunable mechanical properties that match the target tissue environment and provide sufficient structural integrity. In addition, controlled crosslinking behavior and permeability are important to enable construct stabilization and nutrient diffusion [38]. Optimal bioinks should transition from a liquid to a gel state quickly and efficiently, minimizing processing time [39]. There are three main categories of bioink including natural bioinks, synthetic bioinks, and hybrid bioinks.

Natural bioinks include agarose, alginate, cellulose, chitosan, collagen, decellularized extracellular matrix (ECM), dextran, fibrin, gelatin, gellan gum, hyaluronic acid, Matrigel, and silk [40]. These biomaterials closely mimic the ECM, providing a supportive environment for cell growth due to their self-assembling behavior, biocompatibility, and biodegradability [41,42]. However, they often lack the mechanical strength needed to maintain structural stability in vivo and may behave unpredictably [43]. This weakness can lead to printing challenges, fragile tissue constructs, and insufficient cellular support. In addition, these natural materials often show low reproducibility, unstable performance, and high cost [44].

In contrast to natural bioinks, synthetic bioinks provide customizable mechanical strength and controlled degradation. But they typically lack essential biological cues, which can hinder cell activity and limit effective integration with native tissues [45,46]. Synthetic bioink polymers such as poly(ethylene glycol) (PEG), poly(lactic-co-glycolic acid) (PLGA), polylactic acid (PLA), and polycaprolactone (PCL) are approved by the United States Food and Drug Administration (FDA) for use in medical devices due to their biocompatible and biodegradable properties [47]. However, synthetic bioinks require functionalization to be effective in biological applications, enabling cell–polymer interactions, guiding cell behavior, and supporting ECM or tissue remodeling [48].

Hybrid bioinks are composite materials that combine both natural and synthetic components, often with added nanomaterials or crosslinking agents, to leverage the strengths and minimize the weaknesses of each type. The development of hybrid bioinks offers a practical approach to combining the beneficial properties of their components [49]. An ECM-mimetic hyaluronic acid bioink with potassium iodide (KI)-catalyzed crosslinking enables 3D printing of shape-memory scaffolds that can be delivered via minimally invasive injection while supporting stem cell differentiation and cartilage regeneration [50]. A 3D-printed hybrid granular hydrogel ink enabled a light-responsive soft actuator with reversible NIR-triggered bending for programmable soft robotics [51].

The characteristics of the three categories of bioinks are summarized in Table 1.

The three categories of bioink have been used in many medical applications. Natural bioinks offer substantial advantages for skin repair and regeneration, demonstrating a strong capacity to replicate the 3D microenvironment of native skin tissue while supporting cell adhesion, proliferation, migration, and mobility [52]. The use of collagen as a bioink in tissue and organ 3D bioprinting has been significantly limited by its low viscosity and rapid degradation. To overcome these challenges, collagen is commonly combined with other natural biomaterials such as alginate, fibrin, agarose, and hyaluronic acid to enhance its viscosity, stability, and printability [53]. Silk-based bioinks have broad medical applications, including cartilage, bone, vascular, skin, and blood vessel tissue engineering [54]. Alginate-based constructs have been extensively explored for a wide range of applications, including the regeneration of various tissues, the delivery of low molecular weight drugs, wound dressings, and cell encapsulation [55].

PEG derivatives, including hydroxyl, thiol, amine, carboxyl, and acrylate groups, have been employed in 3D printing technologies to enable rapid polymerization. Among them, acrylated PEGs, such as PEG diacrylate (PEGDA) and PEG tetraacrylate (PEGMA), exhibit excellent UV-induced photoreactivity and have been extensively applied in photopolymerization-based techniques [56,57]. PEGDA-based Bioinks with adjustable swelling behavior, mechanical compliance, and photo-reactivity have been developed to improve structural integrity and cross-platform printability across SLA, DLP, and extrusion systems [58]. PCL has become a widely utilized scaffold material in bone tissue engineering due to its degradation rate being comparable to the rate of new bone formation [59]. PLA is well-suited for creating customized orthoses, prostheses, and dentures that demand high precision, as it effectively binds with inorganic bone powder to produce lightweight prosthetic models [60]. Additionally, its semi-crystalline nature allows the formation of nano-scale surface features on 3D-printed bone scaffolds by selectively etching the amorphous regions [61].

A nanoengineered ionic covalent entanglement bioink for 3D bioprinting of patient-specific bone tissues was developed [62]. Nanosilicates within the bioink play a crucial role in stimulating bone-related gene expression. This bioink supports long-term cellular remodeling and promotes endochondral differentiation of human mesenchymal stem cells without the need for growth factors. It enables the fabrication of implantable scaffolds for personalized bone defect repair, advancing 3D bioprinting toward clinical applications in regenerative medicine. A hybrid bioink composed of alginate and cellulose nanocrystals was employed to fabricate a liver-mimicking honeycomb 3D structure embedded with fibroblasts and hepatoma cells [49]. The encapsulated cells maintained high viability throughout the printing process.

## 3. Medical Application of 3D Bioprinting

**Figure 3:**
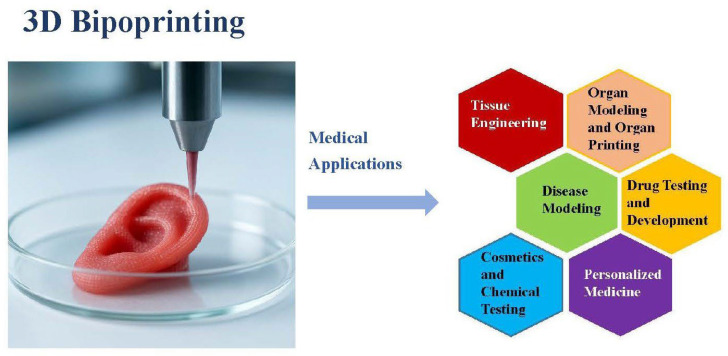
Medical applications of 3D bioprinting (the figure was created by the author).

Several medical applications of 3D bioprinting are discussed in this section (Figure 3).

### 3.1. Tissue Engineering

Tissue damage or injury represents a major health concern, contributing to nearly half of global healthcare expenditures each year [63]. Three-dimensional bioprinting is an emerging additive manufacturing technology to replicate and restore the natural ECM of human tissues and organs [64]. The ECM plays a vital role in regulating cell fate by providing structural support for cell attachment, growth, and proliferation.

Three-dimensional artificial skin models offer versatile platforms for applications such as skin transplantation, the study of disease mechanisms, and the evaluation of biomaterials for skin tissue engineering. Despite these advancements, accurately replicating physiological complexities such as the neurovascular system with living cells in a layered skin structure remains a significant challenge. To address this issue, full-thickness skin models have been fabricated using a DLP 3D printer with methacrylated silk fibroin and gelatin, incorporating keratinocytes, fibroblasts, and vascular endothelial cells to mimic the epidermal and dermal layers [65]. In addition, a functional living skin was successfully developed using a newly formulated biomimetic bioink and DLP-based 3D printing, which supported long-term cell viability and enhanced cell migration and proliferation [66].

Creating vascular networks within engineered tissues remains one of the most significant challenges in biomaterials and tissue engineering. The intricate architecture of these networks has historically hindered their successful fabrication in in vitro tissue models [67]. Recent advancements in biofabrication technologies, including extrusion, laser-based methods, micro-molding, and electrospinning, have improved the ability to construct complex tissue architectures through sequential layering. At the same time, innovative strategies such as the use of decellularized ECMs, self-organizing systems, and cellular sheets are being explored as alternatives to traditional scaffold-forming biopolymers. [68].

Three-dimensional bioprinting enables the integration of cells and biological signals during fabrication, resulting in biologically functional implants. Bioprinting techniques offer extensive potential for creating personalized cartilage constructs, with biological activity being a crucial factor for their regenerative success [69]. A notable application involving cartilage in plastic and reconstructive surgery is nasal reconstruction. Nose-shaped hydrogel constructs were bioprinted using chondrocyte-laden alginate/gellan [70]. These constructs maintained high shape fidelity and secreted cartilage-specific ECMs during in vitro culture.

Repairing critical-sized bone defects remains a major clinical challenge. Bone tissue engineering has gained considerable interest for developing innovative constructs aimed at restoring, preserving, or enhancing bone function [71,72]. To address the limitations of traditional bone grafts, bone tissue engineering using 3D bioprinted scaffolds has emerged as a promising strategy for bone regeneration [73,74]. Functional control modules have been integrated into cell-laden scaffolds through 3D bioprinting [75]. The microarchitecture of bone mesenchymal stem cell (BMSC)-laden methacrylamide gelatin scaffolds was precisely tuned at the micrometer scale using a customized 3D printer. Furthermore, imine-crosslinked S-CHI/A-HA hydrogels have been successfully extrusion-printed into customized scaffolds, where graphene oxide and carbon nanotube fillers modulated mechanical strength, rheology, and printability, revealing a functionalization-dependent trade-off with self-healing capacity [76].

### 3.2. Organ Modeling and Organ Printing

Physical organ models replicate patient-specific anatomy and play key roles in diagnosis and treatment. Three-dimensional printing offers great potential for producing these models by overcoming traditional limitations. However, their clinical use remains limited due to high costs, low fidelity, and insufficient accuracy [77].

A study was conducted to systematically evaluate cardiac MRI (CMR) imaging and segmentation methods for creating accurate virtual 3D heart models for printing [78]. CMR data from nineteen patients were used to generate 76 models through combinations of myocardial and blood pool segmentation. Three models were successfully printed using desktop 3D printers.

As the number of adults with congenital heart disease and heart failure increases, the use of ventricular assist devices remains limited due to complex anatomy and physiology. To improve their application, advanced imaging methods should be explored. Three-dimensional printing can provide patient-specific anatomical models to support pre-surgical planning for cannula and device placement in this population [79].

### 3.3. Drug Testing and Development

The process of drug discovery involves multiple stages, including pre-clinical and clinical testing. Bringing a drug successfully to market demands significant time, effort, and resources, with failures in the later stages often leading to major setbacks. In drug testing, traditional two-dimensional (2D) models grow cells in flat layers, which do not accurately represent the complexity of real human tissues. In contrast, 3D models are designed to more closely replicate the structure and function of actual tissues, resulting in improved performance. As testing methods have advanced, the move from 2D to 3D modeling represents a significant step forward in technology [80,81].

Over the past decade, spheroid technology and micro-organoids have advanced to offer a 3D cellular environment that more closely resembles native tissue. However, organoids used in drug testing are typically only 50–100 μm in size, making them too small to accurately replicate tissue microenvironments, including limitations in nutrient and oxygen supply. A promising alternative is 3D bioprinting, which enables the creation of human tissue equivalents from the ground up, incorporating hollow structures for perfusion and providing precise spatial and temporal control over the placement of cells and ECM proteins [82].

### 3.4. Disease Modeling

There is an increasing demand for biomimetic human tissue models that can provide deeper insights into the pathophysiological mechanisms underlying disease onset and progression. Conventional 2D in vitro assays and animal models have significant limitations in replicating key features of human physiology. Although 3D tissue models offer improved physiological relevance, they are often limited by low throughput and lack essential native-like structural complexity. The recent advancement of bioprinting technologies presents a promising approach to overcoming these challenges by enabling the fabrication of 3D tissue constructs using tailored bioinks, patient-derived cells, and precisely organized biomaterials [83].

Tumor development occurs within a highly dynamic and diverse microenvironment, consisting of multiple cell types and a surrounding ECM. Three-dimensional bioprinting enables the fabrication of intricate, multi-cellular constructs with precisely tunable matrix properties, including composition and stiffness, tailored to specific tumor models. This technology offers a promising approach for accurately replicating the in vivo tumor microenvironment [84].

### 3.5. Cosmetics and Chemical Testing

Assessing the toxicity of new compounds is a vital step in both drug and cosmetics development to ensure product safety and regulatory compliance [85]. Toxicology testing has undergone a major transformation, shifting from traditional reliance on animal models to the widespread adoption of in vitro testing approaches. Integrating 3D bioprinting with microfluidics may transform toxicology testing by improving standardization, accuracy, real-time monitoring, and screening efficiency [86].

With growing interest in non-animal testing methods and the need for more accurate assessments of skin reactions to cosmetic products and chemicals, demand for advanced skin biofabrication technologies continues to rise [87]. Three-dimensional-bioprinted, bioengineered skin substitutes are increasingly used in cosmetics and chemical testing as reliable in vitro models. Tissue engineering generally follows two fundamental strategies: the bottom-up and top-down approaches. The bottom-up approach involves assembling tissues from small cellular building blocks, while the top-down approach begins with a pre-formed scaffold into which cells are seeded. Fabricating a biomimetic, fully functional skin substitute requires leveraging the strengths of both strategies. With the advent of 3D bioprinting, this integration has become feasible. A hybrid bioprinting system that incorporates key elements of both approaches can effectively bridge this gap and enable more precise and functional tissue constructs [88].

By incorporating human-derived cells and customizable bioinks, these 3D bioprinting models offer enhanced biological relevance compared to traditional 2D cultures or animal testing. As regulatory bodies increasingly advocate for ethical and accurate alternatives, bioprinted skin constructs are becoming a valuable platform for safety screening and efficacy testing in pharmaceutical, cosmetic, and toxicological research.

### 3.6. Personalized Medicine

Personalized medicine holds great promise for transforming the healthcare landscape by tailoring treatments to the unique physiological characteristics, drug responses, and genetic profiles of individual patients. Among the emerging technologies driving this shift away from the conventional “one-size-fits-all” approach, 3D printing stands out as a key enabler of personalized medical solutions [89]. The design of personalized implants tailored to the specific dimensions of a patient’s tissue or anatomical cavity has become increasingly important in modern medical practice.

With 3D printing technology, solid dosage forms can now be fabricated by combining multiple drugs into a single unit to enhance patient compliance, facilitate swallowing, or create tailored formulations when standard medications are unavailable [90]. In particular, 3D-printed polypills enable personalized, multi-drug oral formulations with tunable release profiles [91]. Nevertheless, regulatory approval, large-scale manufacturing, and long-term stability require further investigation before widespread clinical adoption.

Beyond oral dosage forms, 3D printing has also advanced the development of implantable medical devices and parenteral systems. Metallic implants and cardiovascular stents can be precisely customized to match patient-specific anatomy. Vat polymerization techniques offer the highest printing resolution among all 3D printing methods, making them highly promising for the fabrication of personalized implants with precise anatomical conformity [92]. Three-dimensional printing has emerged as a viable approach for producing hydrogel-based wound dressings with customizable drug dosages and structural designs [93]. Tailored architectures can be engineered to precisely control the release profiles of therapeutic agents into the surrounding environment. The versatility and adaptability of 3D printing technology enable the production of patient-specific implants for glaucoma and cataract treatment, using biomaterials selected to meet individual clinical needs [94].

The manufacturing of contact lenses is already well established, but the adoption of additive manufacturing presents new opportunities for innovation and personalization. Alam et al. demonstrated the fabrication of personalized smart contact lenses using 3D printing technology, highlighting several advantages over conventional lenses [95]. The process involves generating 3D models through computer-aided design based on standard commercial lens dimensions, selecting suitable materials, and fabricating the lenses using 3D printing techniques.

## 4. The Importance of 3D Printing in Plant-Inspired Application

### 4.1. The Nanomicroscale and Folded Structures of Plant Surfaces

Plant surfaces exhibit highly sophisticated hierarchical architectures that span from the nanoscale to the microscale and macroscale, forming complex folded and multi-level structures that are essential for their remarkable functional performance [96]. Unlike artificial flat surfaces, plant surfaces are characterized by intrinsic three-dimensional topographies composed of ridges, grooves, folds, papillae, trichomes, and layered cuticular features, which together generate unique physicochemical properties [97].

Plants exhibit a remarkable diversity of surface architectures, among which superhydrophobic surfaces are of particular interest for biomimetic applications such as self-cleaning, drag reduction, and surface protection [98,99,100]. These functionalities primarily arise from hierarchical surface structuring, in which hydrophobic wax coatings act synergistically with multi-scale surface features to generate superhydrophobic behavior [101,102]. A representative example is the rose petal, whose surface exhibits a highly sophisticated hierarchical architecture formed by the integration of microscale epidermal papillae and nanoscale waxy protrusions [103]. The micrometer-sized papillae provide a three-dimensional topography that elevates water droplets above the surface, while the nanoscale wax structures further modulate interfacial interactions by reducing the effective solid–liquid contact area. This multilevel organization enables the unique wetting behavior of rose petals. Beyond the rose petal, similar hierarchical principles are widely observed in plant surfaces. Well-known examples include the lotus leaf with extreme water repellency and self-cleaning behavior, and the rice leaf with anisotropic wetting and efficient directional water transport [101].

Plant surfaces exhibit cuticular folds across nearly all above-ground organs, with particularly frequent occurrence on flower petals and seed surfaces [104]. Through controlled folding, wrinkling, and surface undulation, plants dramatically increase effective surface area while maintaining structural integrity. In some plants, for instance, folded epidermal structures and hierarchical trichomes enhance moisture harvesting from fog, whereas in aquatic plants such as Salvinia, multiscale surface structures stabilize air layers under water, enabling persistent drag reduction and gas exchange [105,106].

On Earth, the approximately 10 million extant species display an extraordinary diversity of materials and structural designs, many of which arise through self-assembly and are constructed from a limited set of molecular building blocks [96]. Biological systems possess more complex hierarchical structures and functions than abiotic natural surfaces, and in nature, superhydrophobicity occurs only in living organisms [96]. These hierarchical designs provide valuable models for developing artificial self-cleaning and drag-reducing surfaces [102].

### 4.2. Superiority of 3D Printing Technologies for Bioprinting Applications in Structural Complexity

In the context of 3D bioprinting, the ability to fabricate complex, folded, and hierarchical architectures is critically dependent on advances in 3D printing technologies. In plants, folded and wrinkled surface architectures spanning the nano- and microscale are ubiquitous design strategies used to maximize surface area, regulate mechanical stress, control wettability, and modulate biological interactions. Importantly, such nanomicroscale and folded architectures cannot be adequately reproduced using conventional planar fabrication techniques [107,108]. Traditional top-down manufacturing typically generates simplified or isolated surface features but fails to integrate multi-level, three-dimensional folding with nanoscale precision and spatial continuity. In contrast, advanced 3D printing technologies, particularly high-resolution additive manufacturing and biofabrication platforms, enable the faithful reproduction of plant-inspired folded surfaces by simultaneously controlling geometry, material distribution, and hierarchical organization across multiple length scales [109].

Production volume, degree of customizability, and geometric complexity are key factors in determining whether additive manufacturing or conventional manufacturing methods are most appropriate for a given product [110]. Additive manufacturing enables the deliberate design of complex, hierarchical folding architectures with full three-dimensional control, more closely resembling the multiscale folded structures observed in plant surfaces [105]. While traditional manufacturing techniques are capable of producing folded features, additive manufacturing allows folding to be explicitly designed, controlled, and systematically optimized. Consequently, the nano- and microscale folded architectures found in plant surfaces serve not only as fundamental biological design templates but also as a compelling rationale for adopting 3D bioprinting technologies in plant-inspired surface engineering.

### 4.3. Plant-Inspired Medical Application

**Figure 4:**
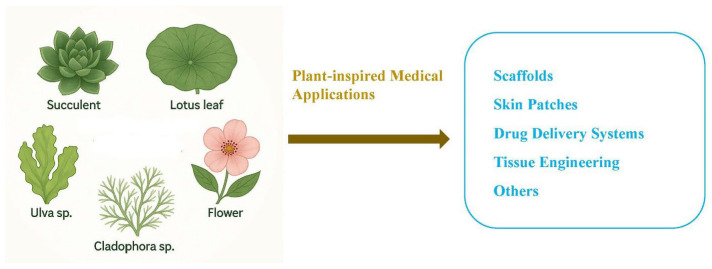
Plant-inspired medical applications (the figure was created by the author).

Many plant-inspired medical applications have been developed through 3D bioprinting. Biomimetic materials with complex plant-inspired structures play an important role in tissue engineering (Figure 4).

Succulent plants are known for their distinctive forms and ability to endure harsh environments, thanks to their specialized structural adaptations. Drawing inspiration from these features, bioceramic scaffolds that mimic succulent plant architectures using digital laser 3D printing of magnesium silicate (MgSiO_3_) were engineered [111]. Unlike conventional scaffolds, these biomimetic designs effectively prevent cell loss and enhance cellular adhesion. By fine-tuning parameters such as leaf dimensions, morphology, and spatial configuration, the internal structure of the scaffolds can be precisely controlled. This results in improved cell loading efficiency, uniform distribution, and enhanced cellular interactions, thereby facilitating the osteogenic differentiation of bone marrow stem cells. In vivo experiments further demonstrated accelerated bone repair, with increased new bone formation.

Skin patches hold significant promise for wound healing and are rapidly evolving into advanced tools for routine health monitoring, offering simplicity and ease of use [112,113]. Initially designed for transdermal drug delivery, skin patches have evolved into multifunctional wearables with integrated sensors, actuators, processors, and communication systems. A wide range of biomedical patches, such as adhesives and hydrogels, have been engineered for effective wound care. A multifunctional, flower-patterned liquid metal (LM)-based hybrid hydrogel scaffold has been developed using 3D printing technology for the efficient treatment of infected wounds [114]. A facile strategy was employed to fabricate antiadhesive and antibacterial gauze featuring a lotus leaf-inspired structure [115]. By integrating hydrophobic coatings and silver nanoparticles, the gauze mimics the lotus leaf’s surface to reduce secondary injury and prevent bacterial adhesion, thereby avoiding delays in wound healing [116].

Drawing inspiration from the layered opening and compartmentalization observed in plant structures, a multi-compartmental capsule was designed for controlled drug delivery [117]. This structure consists of sequential compartments, each defined by gradients in the thickness of both the outer shell and internal partitions. Inspired by shape-changing behaviors in plants, 4D-printed composite hydrogels were developed with programmable, anisotropic swelling guided by cellulose fibril alignment [118]. These structures mimic natural motions and transform into complex 3D shapes when immersed in water, with potential applications in areas like drug delivery, soft robotics, and tissue engineering.

A PVA-MA/GelMA blend hydrogel was used to 3D print complex structures, including a flower with channels, using DPL [119,120]. The flower shape, with its interconnected channels, enhanced cell interaction and supported stem cell-derived osteogenic and chondrogenic tissue formation, demonstrating its potential for advanced tissue engineering applications.

Plant-based skeletons, characterized by their diverse and intricate hierarchical architectures composed mainly of cellulose, hemicellulose, and lignin, offer biocompatible and sustainable platforms with tunable mechanical properties. They have been used in tissue engineering, drug delivery, and biosensing [121]. Marine macroalgae species Ulva sp. and Cladophora sp. were selected as potential ECM alternatives due to their distinct structural characteristics, with Ulva exhibiting a porous architecture and Cladophora a fibrous one [122]. They were evaluated for their suitability as ECM-mimicking scaffolds. Decellularization refers to specialized techniques used in tissue engineering and regenerative medicine involving the removal of cellular components from tissues and organs, leaving behind an acellular scaffold primarily composed of the ECM [123]. In recent years, decellularization techniques have gained widespread use in the development of scaffolds for regenerating damaged organs. Compared to synthetic materials, decellularized ECMs provide distinct advantages by preserving the native microenvironment essential for cellular function and tissue integration [124]. Researchers have extended decellularization methodologies to encompass plant tissues, aiming to exploit the advantages of their structural properties [125,126].

## 5. Rose-Inspired Medical Applications

### 5.1. Medical Applications

The rose is a visually appealing and aromatic plant with diverse medicinal and practical uses [127]. Beyond its aesthetic appeal, the rose has long held symbolic significance in Christian religious history and iconography, symbolizing purity, sacrifice, divine love, and spiritual perfection [128]. Red rose petals were used as natural templates to fabricate osteon-like scaffolds for bone tissue engineering [129]. By combining the petal structure with nanocrystalline forsterite and annealing at 1100 °C, researchers replicated key osteon features such as lamellae, lacunae, and haversian canals. The resulting scaffolds had suitable pore sizes (13–20 μm), showed good bioactivity and biocompatibility, and demonstrated the potential of rose petals in scaffold design.

A bio-inspired implantable anchor device for tendon repair, drawing from rose thorns and limpet teeth to optimize barb design, was developed [130]. Through simulations and tensile tests on swine tendons, the device showed improved load distribution over traditional sutures. However, it still revealed design limitations. The findings highlight the potential and challenges of bio-inspired solutions for tendon repair, paving the way for future improvements.

Although ceramics offer excellent biocompatibility and abrasion resistance, they have largely been replaced by metal-based implants due to their fragility. If their brittleness can be overcome, ceramics would be more suitable for long-term implant use. A rose-inspired ceramic scaffold for bone implants was developed, enhanced with a dual-layer coating of isocyanate (ISO) resin and nano-ZnO [131]. The ISO layer improved mechanical strength by 2–3 times, while the nano-ZnO provided antibacterial properties. In vivo tests demonstrated good biocompatibility with no significant damage observed in major organs, indicating strong potential for safer and long-term bone implant applications.

The limited ability of corneal endothelial cells (CECs) to proliferate and the global shortage of donor tissues highlight the urgent need for effective in vitro CEC expansion methods. To address the limited proliferation and donor availability of CECs, a biomimetic substrate inspired by the hexagonal patterns of white rose petals was developed, which resembles corneal cell structure and density [132]. Using polydimethylsiloxane (PDMS) with rose petal-like topography and cornea-compatible stiffness, functionalized with collagen IV and hyaluronic acid, they achieved improved CEC growth and phenotype retention. This platform offers a promising alternative for corneal therapies, drug testing, and organ-on-chip applications.

Electronic skin (e-skin) is engineered to mimic the sensory and mechanical characteristics of human skin, while also integrating advanced features beyond natural capabilities. Roses have been explored for the development of e-skin technologies [127]. In particular, rose petals and leaves serve as dielectric layers in capacitive-type e-skin sensors [133]. Devices incorporating fresh rose petals demonstrate higher capacitance compared to those using naturally dried petals. Sensors made with dried rose leaves exhibit lower average sensitivity than those with rose petals, due to reduced porosity and smoother surface texture. Elastomeric petals replicated directly from natural rose petals offer a novel and versatile platform for stretchable and printable electronics [134]. Unlike conventional flat PDMS substrates, these biomimetic surfaces effectively suppress the propagation of microcracks in the overlying conductive layer, regardless of the material or deposition technique used. A high-performance flexible e-skin was developed using Cu–Ag core–shell nanowires for enhanced conductivity and oxidation resistance [135]. To boost sensitivity, the microstructure of rose petals was replicated onto a PDMS surface, enabling rapid response, low detection limits, and stable performance for applications such as voice recognition and health monitoring.

Inspired by the unique surface structures of rose petals, PDMS hydrophobic films with inverted petal-like microstructures were fabricated through nanoimprint replication, exhibiting superhydrophilicity combined with high water adhesion [136]. These films enabled stable droplet handling, even when inverted, and were integrated into an acoustic droplet-based platform for forming bladder tumor organoids. The system rapidly produced viable organoids that retained immune components, supporting personalized immunotherapy by inducing tumor-reactive T cells in vitro.

Nanoparticles are promising vehicles for targeted drug delivery due to their nanoscale dimensions [137,138]. To function effectively, these carriers must ensure drug stability during circulation, achieve selective accumulation at the target sites, enable cellular uptake, and facilitate controlled intracellular drug release. An approach for synthesizing spherical boron nitride nanoparticles (BNNPs), with diameters ranging from 100 to 200 nm and characterized by distinct petal-like surface morphologies using chemical vapor deposition, was presented [139]. These uniquely structured BNNPs demonstrated a high drug-loading capacity and exhibited potent cytotoxic effects against tumor cells. These studies suggest petal-like surfaces can be beneficial in drug delivery systems.

Mo et al. investigated how structural defects and high droplet impact velocities influence the wetting behavior of superhydrophobic surfaces, focusing on the transition from lotus-like to petal-like effects [140]. By introducing macro-scale defects inspired by rose petals, they found that droplet adhesion increases under dynamic conditions due to greater energy dissipation and prolonged contact time. These findings offer insights for designing defect-engineered surfaces with controllable droplet retention, useful for applications in droplet manipulation and surface engineering.

### 5.2. The Potential of Rose-Inspired 3D Bioprinting

**Figure 5:**
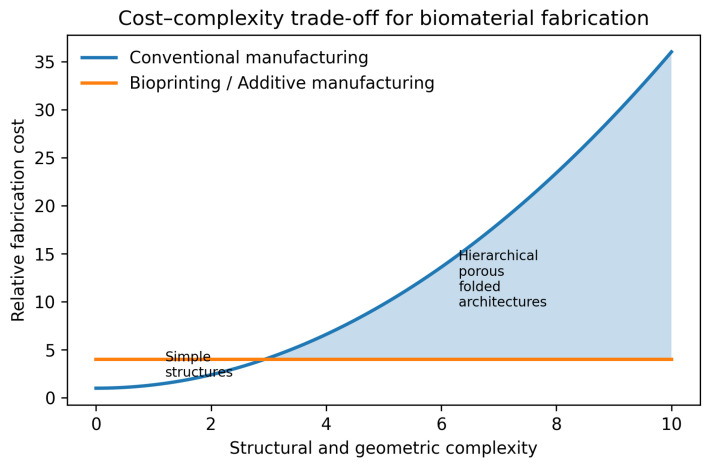
Comparison of cost–complexity trends between conventional manufacturing and bioprinting for hierarchical biomaterial architectures.

Rose-inspired 3D bioprinting in medical applications is reviewed in Section 5.1. Building on these findings, this study suggests even greater potential for applying rose-inspired designs in bioprinted scaffolds, wound dressings, drug delivery systems, and other innovative biomedical applications. From an application-oriented perspective, the hierarchical and folded geometries observed in rose petals can be translated into practical 3D printing design strategies, enabling controlled modulation of mechanical properties, surface interactions, and biological performance. Unlike conventional planar fabrication methods, additive manufacturing allows these complex architectures to be deliberately designed and reproducibly fabricated, thereby facilitating the transition from biomimetic concepts to functional biomedical constructs [141,142,143]. Figure 5 highlights the increasing fabrication burden of conventional manufacturing with structural complexity, in contrast to the relative complexity tolerance of bioprinting and additive manufacturing for producing hierarchical biomimetic architectures.

In scaffold design, rose-inspired architectures can be implemented either as three-dimensional porous frameworks or as engineered surfaces that support cell growth. For example, a spiral, petal-like porous scaffold provides a large surface area and interconnected pore networks, which are beneficial for osteoblast adhesion, vascular infiltration, and new bone formation. Such designs illustrate how rose-inspired geometry can simultaneously enhance structural integrity and biological functionality in bone tissue engineering.

Rose-inspired surface patterning also offers advantages for engineered substrates. Hexagonal microstructures derived from rose petals closely resemble the morphology and density of corneal endothelial cells. PDMS substrates patterned with rose petal-like topography have demonstrated enhanced cell adhesion, monolayer formation, and phenotype retention in corneal endothelial cell cultures [132]. Therefore, rose-mimicking surface patterns may be leveraged in 3D printing either as molds for shaping bioinks or through direct printing of substrates with petal-inspired microstructures, providing a promising strategy for fabricating biomimetic surfaces in tissue engineering applications.

In the development of advanced wound dressings, rose-inspired 3D bioprinting offers promising applications. By mimicking the micro/nano-structured surface of rose petals, which exhibit the petal effect, characterized by a unique combination of superhydrophobicity and high water adhesion [144], engineered wound dressings can retain moisture while protecting the wound from external contaminants. This balance of moisture retention and fluid control is essential for promoting optimal wound healing. Additionally, the increased surface area and hierarchical structure inspired by rose petals can enhance cell adhesion, drug loading capacity, and the controlled release of therapeutic agents, making rose-mimicking bioprinted dressings highly effective in accelerating tissue regeneration and reducing the risk of infection.

For drug delivery applications, rose petal-like structures can be utilized in 3D printing to create bioinspired surfaces capable of manipulating droplets in drug delivery systems. Through advanced 3D printing techniques, researchers can replicate the hierarchical micro- and nanostructures characteristic of rose petals. These printed surfaces can stably retain droplets of pharmaceutical or biological materials, even in inverted orientations, making them suitable for applications such as acoustic droplet platforms, organoid culture systems, and targeted drug delivery patches. Petal-mimicking textures can be fabricated using biocompatible resins, ensuring compatibility with biological environments. Additionally, the printed microstructures can regulate droplet behavior, such as merging, splitting, or movement, enabling controlled and localized drug release. Overall, the integration of petal-effect surfaces into 3D-printed platforms offers significant advantages for droplet manipulation in drug delivery, diagnostics, and broader biomedical engineering applications, representing a convergence of biomimicry and precision manufacturing.

Flowers and plants more broadly exhibit natural self-sealing and self-healing mechanisms that have inspired innovative approaches in biomaterials research. For instance, latex coagulation in Ficus species and callus formation in woody vines demonstrate how plant tissues autonomously repair wounds, sealing off damage before regenerating structural integrity [145]. Rose-inspired materials, when integrated with advanced 3D printing techniques, hold significant potential for developing self-healing biomedical implants. The unique microstructures found on rose petals can be replicated through high-resolution 3D printing to create bioinspired surfaces that not only enhance cell interactions but also enable localized self-repair of minor damage. By combining these rose-like architectures with self-healing hydrogels or polymers, it may be possible to fabricate 3D-printed implants capable of sealing tiny wounds autonomously, thereby improving their durability and functional longevity in tissue engineering and regenerative medicine. The technical challenges and limitations of rose-inspired 3D bioprinting are discussed in the following Section 6, including issues related to printing resolution, cell viability, and industrial scalability.

## 6. Discussion

As 3D printing technology continues to evolve, recent advances have extended its capabilities into the realm of 4D printing, where time becomes a dynamic factor, allowing printed constructs to undergo programmed transformations in response to external stimuli [146].

### 6.1. Technical Challenges and Current Limitations in 3D Bioprinting

Despite these technological advancements, numerous limitations persist within 3D bioprinting. These include restricted bioink options, inadequate vascularization of printed tissues, limited resolution for replicating complex organ structures, and challenges in maintaining long-term cell viability. Additionally, achieving precise spatial organization of multiple cell types and promoting functional tissue maturation are ongoing obstacles.

Many bioinks lack the ideal combination of biocompatibility, mechanical strength, printability, and biological cues needed for personalized applications. Given the wide variety of tissue types in the human body and the complexity of replicating their structural and functional features, numerous bioinks have been explored. However, only a limited number currently exhibit the optimal combination of properties required for effective bioprinting. The development of suitable bioinks must carefully consider a balance of physical, mechanical, and biological characteristics to ensure compatibility with the target tissue [147].

One of the major challenges in 3D bioprinting is maintaining high cell viability throughout the entire process [148]. This includes not only preserving cell health during the printing procedure but also ensuring their long-term functionality after printing. Table 2 provides approximate cell viability ranges and mechanical strength for three bioprinting methods. Another critical issue is sourcing high-quality, patient-specific cells that can endure the mechanical stress of bioprinting while retaining the necessary mechanical and biological functions essential for clinical applications.

Despite advances in scaffold fabrication technology, the in vivo integration and performance of 3D-printed scaffolds remain severely limited due to the lack of effective strategies for promoting vascularization [160]. The absence of robust and reliable methods to induce blood vessel formation within these constructs limits their ability to support cell survival, nutrient exchange, and tissue regeneration after implantation. In response to this critical challenge, researchers over the past decade have actively pursued strategies to enhance vascularization within 3D-printed scaffolds, aiming to improve their biological functionality and facilitate effective integration with host tissues.

Another critical aspect of resolution in 3D bioprinting is the precise spatial positioning of cells within each foundational unit of the printed construct [161]. The proximity and arrangement of cells directly influence physiological outcomes, such as cell signaling and tissue function. Another important factor is shape fidelity, which is the ability of the printed construct to accurately replicate the intended geometry. Various strategies have been developed to enhance the structural accuracy of engineered tissues and organs. As bioprinting progresses toward the fabrication of more complex tissues, incorporating additional functionalities becomes essential. Advancements in data processing, material formulation, and the integration of multiple fabrication techniques are key to replicating the hierarchical architecture of native tissues.

Furthermore, printing resolution at the micro- and nanoscale remains a key limitation in current 3D bioprinting technologies. Although many native tissues and plant-inspired surfaces exhibit hierarchical architectures spanning micro- to nanoscale length scales, most current 3D bioprinting technologies operate predominantly within the microscale regime. Extrusion-based bioprinting is limited by nozzle diameter and bioink rheology, while light-based approaches such as SLA and DLP offer improved resolution but remain constrained by photopolymer diffusion, light scattering, and cell viability requirements. As emphasized in recent high-definition (HD) bioprinting studies [162,163], achieving consistent microscale feature sizes in cell-containing materials remains challenging, and true nanoscale architectural control is typically realized through indirect or hybrid fabrication strategies rather than direct bioprinting.

In addition to biological and structural challenges, industrial viability remains a critical limitation for the clinical translation of 3D bioprinting [164]. The fabrication of complex and highly hierarchical constructs is often associated with prolonged printing times and limited production throughput, which restricts large-scale manufacturing and increases overall cost [165]. Moreover, the need for precise process control, post-printing maturation, and quality assurance further complicates scalability and standardization. Addressing these time- and cost-related constraints will be essential for improving the industrial feasibility of advanced bioprinting technologies.

### 6.2. Implications for Medical and Personalized Applications

Overcoming the limitations of bioprinting is crucial to unlocking its full potential in personalized medicine and regenerative healthcare. Plant- and rose-inspired innovations within these applications may be one of the potential strategies to address these limitations of replicating human tissues. Compared to human tissues, plant tissues are simpler in architecture, more resilient, and capable of surviving in diverse environments. While plant tissues and human tissues are not the same, they have some similarities in biological mechanisms. Therefore, plant tissues have emerged as a valuable source of inspiration to replicate the complicated structural and functional human tissues. These characteristics of plants make them useful for developing bioinspired materials and systems. From ancient times to modern biomedical engineering, plant systems have offered structural and functional cues that can be translated into advanced solutions for tissue repair, regeneration, and drug delivery. This cross-disciplinary approach enriches the field of 3D bioprinting by incorporating naturally evolved mechanisms into modern medical design. In addition, organ donation has sparked ongoing debates, often centered around ethical practices and related concerns. The persistent shortage of deceased donor organs and the limited availability of brain-dead donors have renewed interest in donation after circulatory death (DCD) as an alternative source for transplantation [166]. Controlled organ DCD has been reintroduced in the UK to boost organ donation rates, but has sparked ethical controversy due to its impact on end-of-life care [167]. Tissue engineering seeks to overcome the limited availability of donor organs by developing bioengineered alternatives for transplantation and repair [168]. If 3D printing technology advances to the point where functional organs can be fabricated, it could help address these concerns by reducing dependence on donor organs. While 3D bioprinting itself raises important ethical challenges, such as the definition of life, ownership of bioengineered organs, and equitable access, these technologies can still offer significant benefits to humanity when developed and applied under proper ethical oversight and regulatory control.

Beyond these ethical and clinical considerations, the future development of plant- and rose-inspired 3D bioprinting also depends on advances in manufacturing strategies and materials.

From a technological perspective, advances in extrusion-based bioprinting, photopolymerization-based printing, and hybrid fabrication strategies are expected to enable more precise control over geometry and structural organization, which is critical for replicating plant-inspired ultrastructures. Artificial intelligence (AI) is increasingly transforming 3D printing technologies; the integration of AI algorithms into additive manufacturing systems enables real-time optimization of printing parameters, accurate prediction of material behavior, and early defect detection through computer vision and sensor data [169]. Real-time control of mechanical properties is essential in biomanufacturing. By adjusting process parameters such as printing speed, layer thickness, and scaffold architecture, mechanical performance can be optimized for in vivo stability. Machine learning further enables data-driven design, real-time monitoring, and process optimization [170].

From a materials and manufacturing standpoint, future development of this domain is expected to focus on the translation of plant-derived hierarchical architectures into printable design principles, including multiscale surface topography, controlled folding, and mechanically compliant structures. The integration of AI with nanomaterials is reshaping metal 3D printing for biomedical applications [171]. Advances in polymer chemistry, hydrogel-based bioinks, and multi-material printing are likely to expand the range of biomimetic architectures that can be reliably fabricated [172].

At the application level, the integration of these bioinspired architectures into bioprinted scaffolds, wound dressings, and drug delivery systems may offer new opportunities to regulate cell–material interactions, mechanical compliance, and functional performance.

## 7. Conclusions

Three-dimensional printing is a disruptive technology that continues to redefine both manufacturing and healthcare. Among its most transformative branches, 3D bioprinting has emerged as an advanced additive manufacturing method that uses bioinks, comprising living cells and biomaterials, to fabricate tissue-like structures for diverse medical applications. This review highlights its key uses in tissue engineering, organ modeling and printing, drug testing and development, disease modeling, cosmetics and chemical testing, and personalized medicine.

In parallel, the biomimicry of natural plant structures offers exciting opportunities for innovation in biomedical material design. Among these, the rose is particularly notable for its intricate hierarchical geometry, referring to the multiscale organization of surface microstructures and underlying tissue architectures. Such hierarchical features can be translated into 3D printing design strategies to control surface topography, mechanical compliance, and structural stability in bioprinted constructs. Incorporating rose-inspired architectures into 3D-bioprinted constructs can enhance mechanical robustness, flexibility, and surface conformity, thereby improving the functionality of medical materials.

This paper reviews plant- and rose-inspired approaches in medical applications and explores the potential of rose-inspired 3D bioprinting in advancing functional and biomimetic tissue models. Among the biomedical applications discussed, bioprinted scaffolds, wound dressings, and drug delivery systems emerge as priority areas where rose-inspired biomimicry is expected to have the greatest impact, driven by the critical importance of surface architecture, mechanical compliance, and interactions between cells and materials. Despite existing challenges, ongoing innovation and interdisciplinary collaboration are expected to propel the field forward and broaden its clinical impact.

## Figures and Tables

**Table 1:** Bioink categories and their characteristics.

Category	Materials	Functions	Limitations
Natural bioinks	Gelatin, collagen, alginate, hyaluronic acid, fibrin, agarose, chitosan, cellulose, decellularized ECM, dextran, gellan gum, Matrigel, silk	Biocompatibility, biodegradability, mimic ECM, support for cell adhesion and proliferation	Weak mechanical strength, low printability, rapid degradation, low reproducibility, unstable performance, and high cost
Synthetic bioinks	PEG, PLGA, PLA, PCL, polyurethane	Tunable mechanical properties, controlled degradation, and good printability	Poor cell adhesion, lack of bioactivity, and a need for functionalization or blending
Hybrid bioinks	Combinations of natural and synthetic materials, often with nanomaterials	The integration of the bioactivity of natural components with the mechanical robustness and stability of synthetic materials	Complex formulation, potential compatibility issues, and the need for optimization of crosslinking

**Table 2:** Representative quantitative properties reported for 3D bioprinted constructs using different printing methods and bioink systems.

Bioprinting Method	Cell Viability	Mechanical Strength	References
Vat polymerization	85% to 95%	Relatively high	[149,150,151]
Material extrusion	70% to >90%	Moderate to low	[152,153,154,155,156]
Material jetting	82% to >94%	Low	[157,158,159]

## Data Availability

No new data were created or analyzed in this study. Data sharing is not applicable to this article.
